# Distal mechanisms in robotic surgical instruments: a structured review of surgical access, miniaturisation constraints and procedure-specific performance trade-offs

**DOI:** 10.1007/s11701-026-03929-x

**Published:** 2026-09-09

**Authors:** Carlos E. Díaz Gutiérrez, Víctor Eduardo Quiroz Velázquez, Roemi Fernández

**Affiliations:** 1 Division of Basic Sciences and Engineering, Universidad Autónoma Metropolitana, Lerma Campus, Lerma, State of Mexico Mexico; 2 Centre for Automation and Robotics CAR (CSIC-UPM), Consejo Superior de Investigaciones Científicas, Ctra. Campo Real Km 0,2 La Poveda, Arganda del Rey, Madrid Spain

**Keywords:** Robotic surgery, Distal mechanism, Flexible surgical robot, Robotic endoscopy, Continuum robot, Tendon-sheath transmission, Variable stiffness, Surgical validation

## Abstract

The distal end of a robotic surgical instrument is where access, dexterity, tissue interaction and procedural capability converge. In flexible, endoluminal, transoral, percutaneous, vascular and neuroendoscopic surgery, the clinical value of an instrument depends not only on kinematic complexity but also on miniaturisation, usable lumen, transmission reliability, stiffness, sensing and validation maturity. This article presents a structured narrative review of distal mechanisms for robotic surgical instruments, based on a technical-clinical synthesis of studies identified through IEEE Xplore, PubMed and Web of Science. Distal architectures are organised into discrete articulated, continuous and hybrid mechanisms and are interpreted according to surgical access route, dominant task, tissue interaction requirements and procedure-specific performance trade-offs. The review shows that no single distal architecture is universally superior. Discrete mechanisms can support local orientation and load-bearing tasks; continuous mechanisms facilitate navigation through tortuous anatomy but introduce shape-estimation and transmission challenges; and hybrid architectures seek to balance access, stability, dexterity and deployment capability. Across these families, miniaturisation should be understood as functional rather than dimensional: reduced outer diameter is clinically meaningful only when force transmission, usable lumen, stiffness, sensing and workflow compatibility are preserved. The article proposes a minimum reporting core for distal robotic mechanisms, including outer diameter, usable lumen, transmission principle, distal force, stiffness definition and validation level. Future progress will depend on procedure-specific evaluation frameworks linking mechanism design with surgical task, anatomical constraints and translational readiness.

## Introduction

Robotic surgery is often described through platforms, control systems, imaging technologies or surgeon-robot interfaces. However, in many minimally invasive procedures, the usefulness of a robotic instrument depends on its distal end. This is where anatomical access, local dexterity, tissue interaction, force transmission, sensing and procedural capability must fit within a limited volume. In endoluminal, transoral, percutaneous, vascular, neuroendoscopic and single-port procedures, the distal mechanism must pass through a restricted access route, navigate complex anatomy, avoid damaging healthy tissue and perform a clinically meaningful action at the target site. These actions may include exposure, traction, cutting, coagulation, suturing, grasping, perforation, device deployment or needle guidance [1–9]. The main challenge is therefore not only to move a tip through space, but to provide reliable local surgical capability within a small, deformable and procedure-specific anatomical environment [1, 3–6, 9].

For this review, a distal mechanism is defined as the distal or near-distal component that enables surgical access, local motion, tissue interaction, force transmission, sensing, stabilisation, deployment or task execution at the operative site. An extended distal architecture refers to a configuration in which clinically relevant distal function is distributed across a wrist, continuum segment, sheath, deployable body, active guide, magnetic interface, locking element or delivery system, rather than being confined to a conventional end effector.

The main abbreviations used in the manuscript are defined here: ESD = Endoscopic Submucosal Dissection; NOTES = Natural Orifice Transluminal Endoscopic Surgery; TORS = Transoral Robotic Surgery; OD = outer diameter; DoF = degrees of freedom; NR = not reported or not clearly distinguishable in the consulted source.

This focus matters because mechanically convincing designs may lose clinical viability when considered against workflow, tissue safety, access constraints, instrument exchange and validation maturity [3, 4, 7, 9–11]. A dexterous wrist may be less useful if it occupies the channel needed for vision, irrigation, suction or additional instrumentation [3, 4, 7, 11, 12]. A thin flexible or continuum segment may improve access but lack sufficient traction, distal load or positional stability [7, 13–16]. Variable-stiffness elements may improve stability, but only if diameter, response time, actuation requirements, locking reliability, sterilisation burden and failure modes remain compatible with the intended procedure [3, 4, 13, 17–29]. Distal design should therefore be understood as a procedure-specific balance among access, dexterity, force, stiffness, sensing, tissue safety and translational readiness.

A clinically useful taxonomy of distal mechanisms must go beyond size, DoF or geometric elegance [1, 2, 30]. It should explain how surgical function is achieved under anatomical, mechanical and transmission constraints. In this review, three architectural families are used as implementation patterns for surgical functions. Discrete articulated mechanisms concentrate motion at identifiable joints and may provide local orientation, torque, grasping capability or load-bearing capacity [7, 15, 31]. Continuous mechanisms generate motion through distributed bending and are useful for tortuous anatomical pathways, but must be interpreted in relation to stiffness, load response, shape estimation and transmission nonlinearities [2, 14, 16, 32–34]. Hybrid and extended architectures combine elements such as discrete joints, flexible segments, deployable arms, guide sheaths, magnetic actuation, cable-pneumatic actuation, variable stiffness or shape locking to balance access, stability and tissue-interaction capability [3, 9, 13, 24, 25, 28, 35–38]. These categories are not fixed boundaries, because many clinically relevant devices combine more than one family. Their purpose is to identify the dominant trade-off in each surgical scenario.

Miniaturisation, transmission, sensing and validation maturity are cross-cutting requirements across these families. A smaller OD is clinically useful only when functional capability is preserved, because each millimetre may need to accommodate structure, joints or continuum backbone, tendons or cables, working channel, optics, irrigation, insulation, sensors and stiffness-control elements [3, 5, 7–9, 13, 14, 25, 28]. Similarly, tendon, cable and tendon-sheath mechanisms support remote actuation and flexible access, but introduce friction, elongation, slack, backlash, hysteresis, force attenuation and configuration-dependent errors [33, 34, 39–45]. Distal sensing helps make tissue interaction observable, while validation maturity determines whether mechanical performance can be interpreted in a clinically meaningful way [46–49].

This article presents a structured narrative review of distal mechanisms in robotic surgical instruments. It does not perform a statistical meta-analysis or rank devices using a single benchmark. Instead, it reviews technical and clinically relevant evidence on discrete, continuous and hybrid distal architectures, focusing on miniaturisation, usable lumen, distal force, stiffness, transmission reliability, sensing and validation maturity. The review prioritises surgical function over kinematic complexity. Whereas several prior reviews have focused primarily on mechanism families, robotic platforms, actuation strategies or specific clinical domains, the present review links distal architecture to task-specific surgical interpretability.

This review makes three contributions. First, it proposes a functional taxonomy of distal mechanisms based on surgical use rather than mechanism type alone. Second, it examines trade-offs among OD, usable lumen, force transmission, stiffness, sensing and validation across surgical scenarios. Third, it proposes a minimum reporting core for distal robotic mechanisms: OD, usable lumen or functional channel, transmission principle, distal force or load capacity, operational definition of stiffness and validation level.

To clarify this positioning, Table 1 summarises how the present review differs from prior review areas cited in the manuscript. The key distinction is that the three architectural families are not presented as a new topological classification in themselves; rather, they are used as an organising structure to examine how distal architecture affects surgical access, task execution, usable lumen, force transmission, stiffness, sensing, workflow compatibility, and validation maturity, thereby supporting task-specific surgical interpretability.

**Table 1:** Comparison between previous review areas and the contribution of the present review

Previous review area	Typical emphasis	Gap addressed in this review	Specific contribution of the present review
Continuum and soft surgical robots [2, 32, 50]	Kinematics, actuation, modelling, shape control, and navigation through curved anatomy	Distal performance is not always interpreted in relation to surgical task, usable lumen, force transmission, and validation context	Interprets continuous mechanisms by access route, tissue interaction, stiffness, transmission losses, and task-specific usefulness
Variable-stiffness mechanisms [17–20]	Materials, locking strategies, stiffness modulation, and structural stability	Added stiffness is not always weighed against diameter, workflow, response time, sterilisation, and procedural benefit	Treats stiffness as a functional trade-off linked to access, manipulation, safety, and translational readiness
Flexible and robotic endoscopy [51–53]	Endoscopic platforms, access routes, triangulation, visualisation, and therapeutic task support	Platform level discussion may obscure the specific role of the distal mechanism	Focuses on the distal component as the interface where access, dexterity, sensing, lumen, and tissue interaction converge
Wristed and articulated mechanisms [1, 30]	Joint design, dexterity, DoF, local orientation, and payload	Dexterity metrics may be reported without sufficient attention to internal volume, usable channels, and surgical workflow	Evaluates discrete articulation by what it enables and what it sacrifices inside the distal envelope
Present review	Distal mechanisms in robotic surgical instruments	Need for a surgically interpretable framework linking architecture, task, constraints, and validation	Provides a functional taxonomy and reporting core for OD, usable lumen, transmission principle, distal force, stiffness definition, and validation level

## Review approach and evidence selection

This article is a structured narrative review with explicit technical screening, not a formal systematic review or meta-analysis. It does not pool clinical outcomes, complication rates or therapeutic efficacy. Instead, it synthesises technical and clinically relevant evidence on distal robotic mechanisms, miniaturisation, transmission, variable stiffness, sensing, performance metrics and surgical validation. Studies were interpreted by both mechanism type and surgical function, considering access route, dominant task, usable lumen or functional channel, distal force or load capacity, stiffness behaviour, transmission losses, sensing and validation context.

### Search strategy and eligibility criteria

The literature search was conducted from 20 April to 6 May 2026 in IEEE Xplore, PubMed and Web of Science. The initial broad search string was: ("flexible surgical robot") OR ("robotic endoscopy") OR ("flexible endoscopy robot") OR ("continuum surgical robot") OR ("soft surgical robot"). In IEEE Xplore, the search was performed in the “All Metadata” field. In Web of Science and PubMed, an equivalent search was applied to the available bibliographic fields.

Because the review focuses on distal mechanism design, the seed search was complemented during screening by targeted cross-checking using distal-design descriptors, including wrist, distal, tip, tendon-sheath transmission, variable stiffness, shape locking, magnetic actuation, magnetic catheter, magnetic continuum robot, soft magnetic robot, surgical vision and continuum robot modelling. These descriptors guided eligibility assessment and verification of relevant literature, rather than defining a fully reconstructed systematic search.

Targeted cross-checking also considered representative works on surgical data science, vision-guided endoluminal navigation, robotic magnetic navigation, magnetic capsule endoscopy, magnetic soft and continuum robots, microrobotics, telemanipulated vascular intervention, biomimetic endoscopic devices and continuum-robot modelling [54–72]. These sources were integrated only when they clarified distal architecture, actuation, sensing, localisation, navigation, transmission, miniaturisation, validation context or workflow. Broader works in medical image segmentation or targeted drug delivery were treated as contextual rather than central evidence when they did not directly address distal surgical mechanisms.

Studies were retained when they provided verifiable evidence on distal mechanism design, miniaturisation, tendon or tendon-sheath transmission, variable stiffness, shape locking, distal sensing, performance metrics, functional validation or an explicit surgical scenario [3, 7–9, 13–15, 28, 48, 49, 73]. Studies on computer vision, generic navigation, training, haptic interfaces, orthopaedic robots without distal miniaturisation, industrial mechanisms or proximal support systems were excluded when they did not describe distal architecture or surgical performance. Control or learning studies were used as secondary evidence only when they clarified transmission losses, force estimation, distal precision or performance under load [33, 34, 47, 74, 75].

### Data extraction and synthesis criteria

Extracted information was organised through variables that allow comparison across heterogeneous mechanisms: surgical scenario, access route, distal architecture, OD, usable lumen or functional channel, DoF, transmission principle, distal force or load capacity, stiffness or variable-stiffness behaviour, sensing, accuracy or tracking performance, workspace and validation type.

The synthesis was qualitative and comparative. Pooled statistical analysis was not performed because studies differed widely in architecture, scale, access route, task, validation environment and metrics. Representative cases were examined to identify trade-offs between OD and usable lumen, transmission losses and distal reliability, flexibility and stiffness, and validation metrics and clinically interpretable tasks.

In this manuscript, NR means that information was not reported or could not be clearly identified in the consulted source. It does not indicate design deficiency; it means that the variable could not be used in a verifiable quantitative comparison. Useful lumen and functional channel were treated as different unless the source supported their equivalence.

### Selection flow, traceability and reconstruction limits

**Fig. 1:**
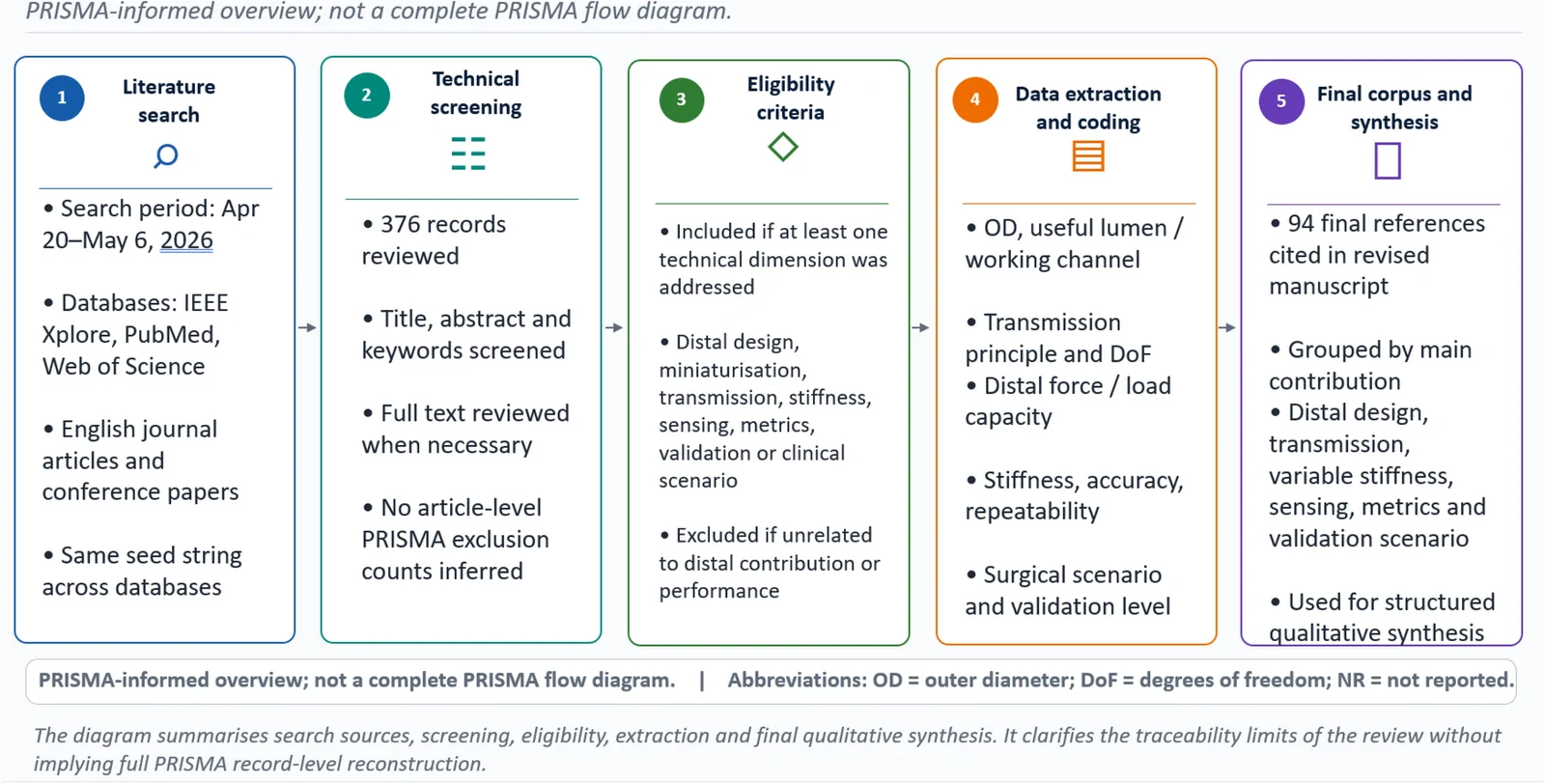
Structured literature selection flow. PRISMA-informed overview of search sources, screening, extraction and synthesis; not a complete PRISMA flow because record-level exclusions and duplicate handling were not reconstructed

Figure 1 presents the selection flow as a PRISMA-informed overview, not a complete PRISMA diagram, because record-level exclusions, full-text retrieval status and duplicate handling were not reconstructed for all records.

The screening matrix documents combined and unique records, cross-database duplicates, candidates by role and stage 2 criteria. No extra duplicates or exclusion reasons were inferred. Table 2 summarises the documented selection flow and indicates which elements were available and which were not reconstructed. This preserves traceability while avoiding unsupported methodological precision.

**Table 2:** Documented elements of the literature selection and screening process

Selection element	Available documented information	Use in this review
Search period	20 April–6 May 2026	Defines the time window of the literature search
Databases searched	IEEE Xplore, PubMed and Web of Science	Defines the technical-clinical evidence sources
Search strategy	Same seed search string applied across databases; targeted technical descriptors used during screening	Ensures consistency of the initial search approach
Publication type	English-language journal articles and conference papers	Defines the eligible publication formats
Records reviewed	376 records reviewed	Forms the basis for title, abstract and keyword screening
Screening level	Title, abstract and keywords; full text when necessary	Supports iterative technical screening
Eligibility focus	Distal mechanism design, miniaturisation, transmission, stiffness, sensing, metrics, validation or explicit surgical scenario	Defines inclusion around distal robotic contribution and surgical relevance
Exclusion focus	Studies unrelated to distal architecture, distal contribution or surgical performance	Defines the conceptual exclusion boundary
Data extraction variables	Surgical scenario, access route, architecture, OD, usable lumen or functional channel, DoF, transmission, force/load, stiffness, sensing, accuracy and validation level	Supports qualitative comparison across heterogeneous mechanisms
Missing information convention	NR used when information was not reported or not clearly distinguishable	Avoids unsupported inference of unreported variables
Final cited corpus	94 references cited in the revised manuscript, including the original technical-clinical evidence base and additional representative works identified during targeted cross-checking	Forms the basis for structured qualitative synthesis and for contextualising distal mechanisms, magnetic actuation, sensing, modelling, validation maturity and translational readiness
Reconstruction limits	Article-level exclusion reasons, full-text retrieval status and duplicate-handling information were not reconstructed for every record	Clarifies why the figure is PRISMA-informed but not a complete PRISMA flow diagram

### Scope limitations

IEEE Xplore, PubMed and Web of Science were appropriate for a technical-clinical review of robotics, mechatronics, instrumentation, sensors, control and medical robotics. However, Scopus and clinical trial registries were not included, and the review should not be interpreted as a comprehensive assessment of clinical efficacy, safety or adoption.

The review was also not designed as an author-team or laboratory-centred mapping exercise. Influential research programmes in vision-guided surgical robotics, computer-aided surgery, magnetic micro- and millimetre-scale robotics, magnetic catheters, biomimetic endoscopic devices and continuum-robot modelling may therefore be underrepresented when their studies did not meet the distal-mechanism and surgical-performance focus of the screening strategy. Additional sources are discussed only when they clarify distal function, magnetic or mechanical actuation, sensing, navigation, modelling, validation or clinical workflow. Future work should include Scopus, clinical trial databases and procedure-specific clinical literature to evaluate safety, complications, usability, learning curves and translational outcomes.

## Functional taxonomy of distal mechanisms

In this review, surgical function is used as the primary interpretive variable and architecture as the implementation route. The classification was therefore applied in two steps. First, each mechanism was interpreted according to the dominant surgical demand it supports, such as constrained access, exposure, traction, local orientation, cutting, suturing, stabilisation, deployment, needle guidance, force-aware tissue interaction or navigation. Second, the corresponding architecture was identified as discrete, continuous, hybrid or extended according to how that function is mechanically realised. In this sense, topology does not determine the classification by itself; it helps explain how a specific surgical function is achieved, limited or validated under anatomical and workflow constraints. Accordingly, the three architectural families should be read as implementation patterns for surgical functions, not as an outcome-independent topological ranking.

A clinically useful taxonomy should not classify devices only by size, shape or DoF [1, 2, 30]. The same mechanism may have different value depending on whether the procedure requires access, exposure, traction, cutting, suturing, stabilisation, deployment or needle guidance. Distal architectures should therefore be interpreted by task and by constraints on usable lumen, force transmission, stiffness, sensing and workflow [3, 5, 7, 9, 51–53].

The literature can be organised into three implementation families. Discrete articulated mechanisms concentrate motion at identifiable joints and are evaluated through orientation, payload, torque, grasping or task execution, as in rolling-contact joints, articulated surgical drills and miniature distal-rotation instruments [7, 15, 31]. Continuous mechanisms use distributed bending and suit curved anatomical pathways, but must be interpreted with stiffness, load effects, shape estimation and transmission nonlinearities [14, 16, 33, 34, 76]. Hybrid or extended architectures combine discrete joints, flexible segments, deployable arms, guide sheaths, magnetic actuation, cable-pneumatic actuation, variable stiffness or shape locking to balance access, stability and tissue interaction [3, 9, 13, 24–26, 28, 36, 38].

**Fig. 2:**
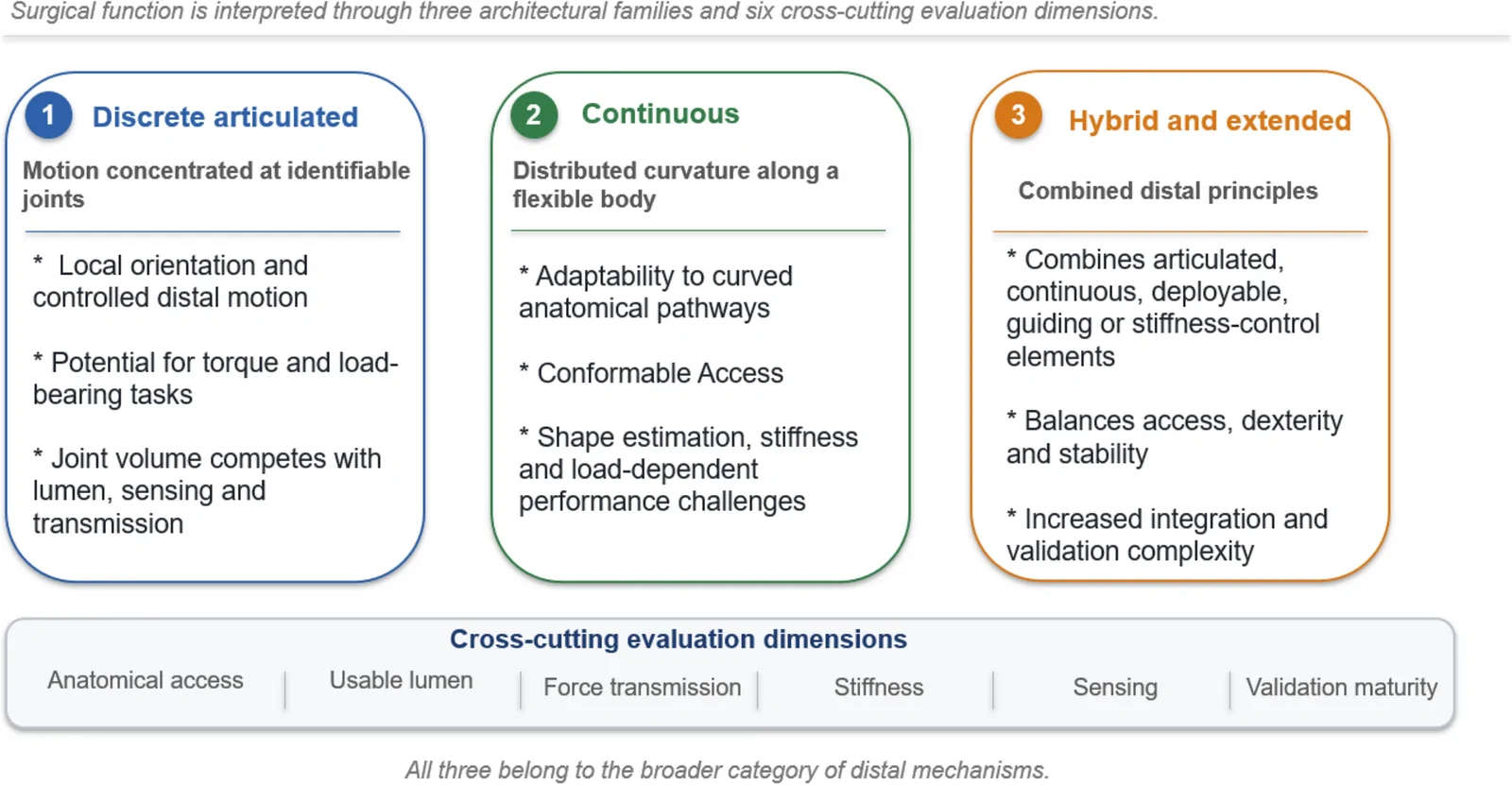
Functional taxonomy of distal mechanisms. Architectural families are interpreted as implementation patterns for surgical functions

These families are not rigid categories. Many clinically relevant devices combine more than one architectural principle, and classification may depend on which component most strongly affects surgical performance. The distinction remains useful because each family succeeds, fails or requires validation for different reasons: discrete mechanisms by volume and integration constraints; continuous mechanisms by shape uncertainty, stiffness and transmission losses; and hybrid architectures by complexity, workflow integration and validation maturity [3, 9, 13, 24, 38]. Figure 2 summarises this classification.

### Discrete articulated mechanisms

Discrete articulated mechanisms concentrate movement at defined joints. Their main advantage is local controllability: orientation, rotation, grasping, drilling or other distal actions can be generated through relatively clear kinematic relationships [7, 15, 31, 77]. This is valuable when the surgical function requires local torque, focal force, repeatability or stable orientation in a confined space, as in articulated forceps, miniaturised drills or neuroendoscopic effectors [7, 15, 31, 77].

**Fig. 3:**
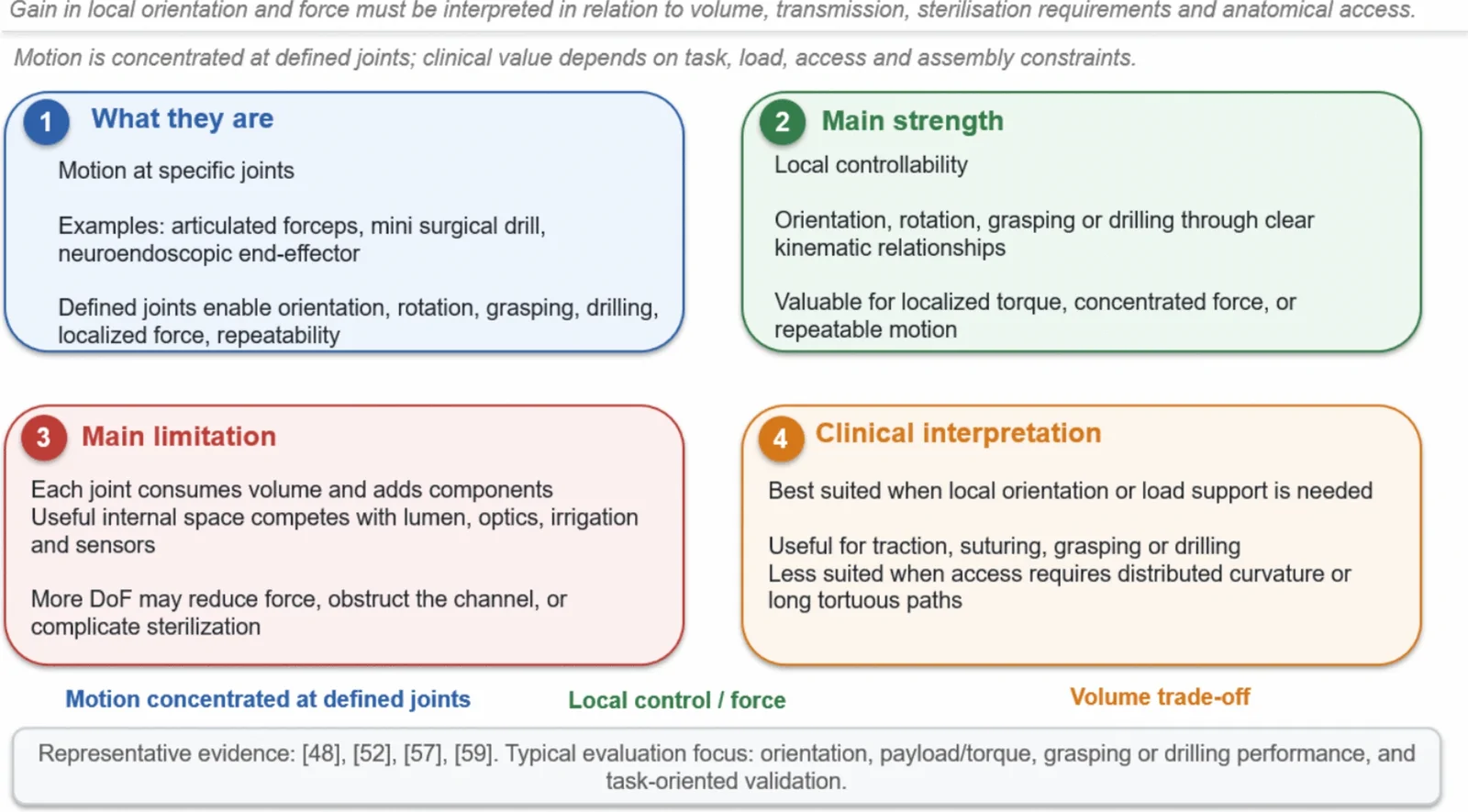
Discrete distal mechanisms. Local orientation and force must be interpreted against volume, transmission and access constraints

Their limitation is that each joint occupies volume and competes with usable lumen, optics, irrigation, sensors, insulation and transmission elements [3, 7, 13, 15, 31]. Discrete mechanisms are therefore strongest when local orientation, load-bearing capacity or controlled manipulation are needed at the target site, but less suitable when the dominant challenge is long curved access, distributed tissue contact or tortuous navigation. In those settings, continuous or catheter-like architectures may follow anatomy better, although they introduce shape estimation, friction, hysteresis and load-dependent control challenges [2, 14, 16, 32–34, 50]. Figure 3 summarises these concepts.

### Continuous mechanisms

Continuous mechanisms generate motion through distributed curvature rather than a sequence of discrete joint rotations [14, 16, 76]. Their principal clinical advantage is not necessarily superior angular precision, but the ability to conform to constrained anatomical routes, reduce local collisions and reach targets that may be difficult or unsafe for rigid articulated chains [14, 16, 76].

Distributed curvature also complicates distal reliability. It becomes harder to determine instrument shape, estimate tip position and orientation, and maintain force or precision under load. These limitations increase when tendon or tendon-sheath transmission adds friction, elongation, slack, backlash, hysteresis and configuration-dependent errors [33, 34, 74]. Continuous mechanisms should therefore be evaluated by stiffness, load response, shape estimation and transmission behaviour, not only by bending range or workspace.

**Fig. 4:**
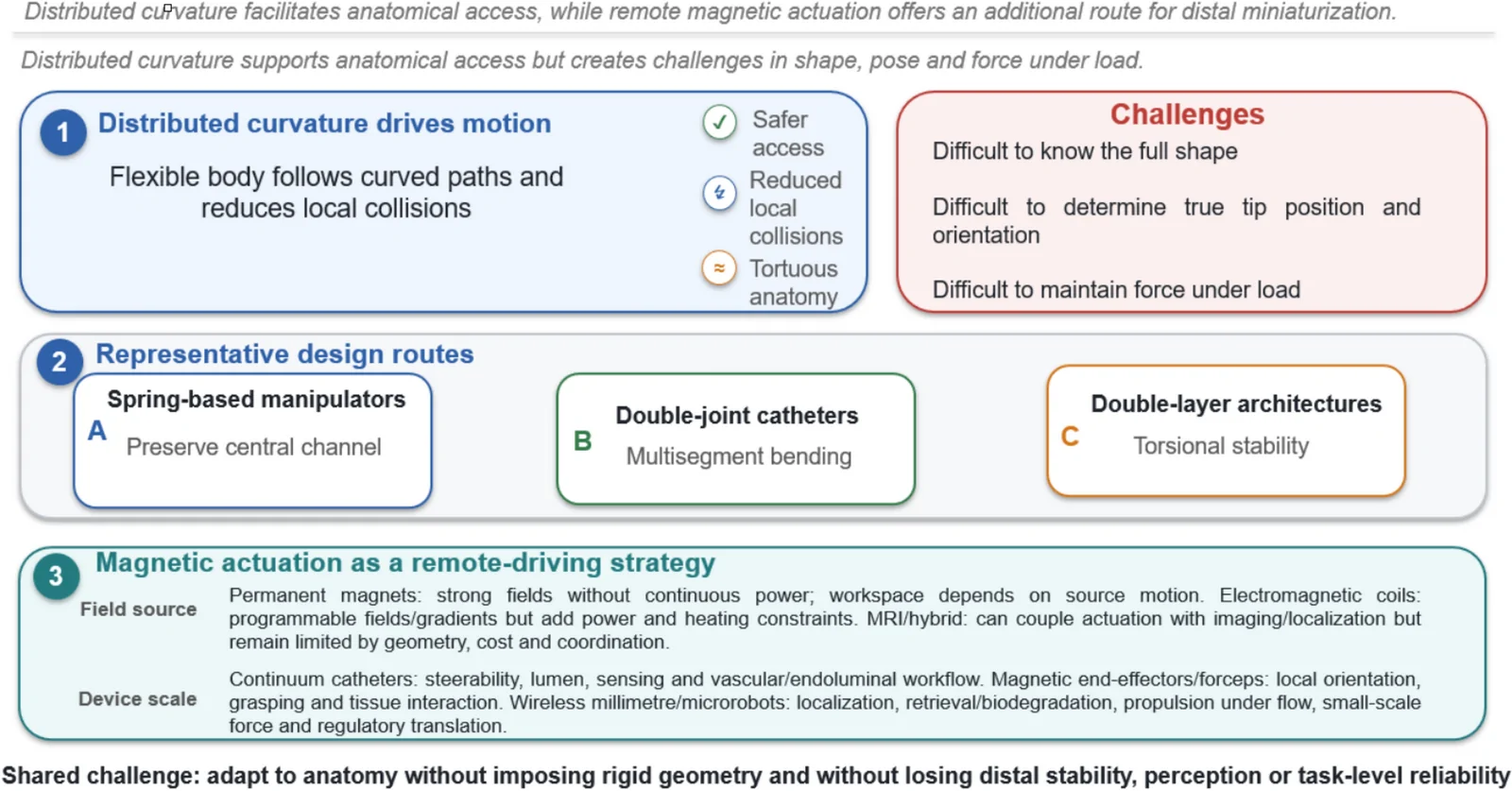
Continuous distal mechanisms. Distributed curvature facilitates access but complicates shape estimation, stiffness control and comparison

Examples include spring-based manipulators that preserve the central canal [14], double-jointed catheter robots with wire-reinforced spring backbones [16], double-layer architectures with superelastic central columns [76], and magnetic actuation that moves part of the drive mechanism outside the instrument [36, 37]. Recent work on parallel-continuum robots, tendon-tension calibration and morphology descriptors further supports the need to interpret continuous mechanisms through modelling, calibration, morphology and repeatability, not only through nominal DoF or bending range [65, 68–70]. Figure 4 summarises continuous distal mechanisms.

### Hybrid and extended distal architectures

Hybrid architectures emerge when neither a discrete chain nor a continuous body alone satisfies the surgical requirements of the procedure. In therapeutic endoscopy, transoral surgery, NOTES, percutaneous access and cardiovascular intervention, the instrument often needs to remain flexible during entry and navigation, but stable during manipulation, exposure, deployment or tissue interaction [3, 5, 9, 38, 77]. This has led to combinations of continuous bodies, discrete wrists, deployable arms, variable-stiffness guide sheaths, pneumatic-cable joints, shape-locking mechanisms and delivery systems with integrated visualisation [3, 9, 13, 24, 25, 28, 38].

This category requires a broader definition of the distal mechanism. The clinically relevant distal component may include structures that enable access, stabilisation, orientation, sensing, triangulation or deployment at the operative site, rather than only a conventional articulated end effector [3, 9, 13, 25, 28, 38]. ValveTech illustrates this broader interpretation: performance is assessed through alignment, stabilisation and deployment capability in an anatomical simulator, not only through kinematic dexterity [9]. Similarly, deployable arms, variable-stiffness sheaths and hybrid pneumatic-cable continuum joints should be interpreted by how they support triangulation, exposure, load capacity, instrument stability or controlled tissue interaction [3, 9, 13, 24, 25, 28, 38].

**Fig. 5:**
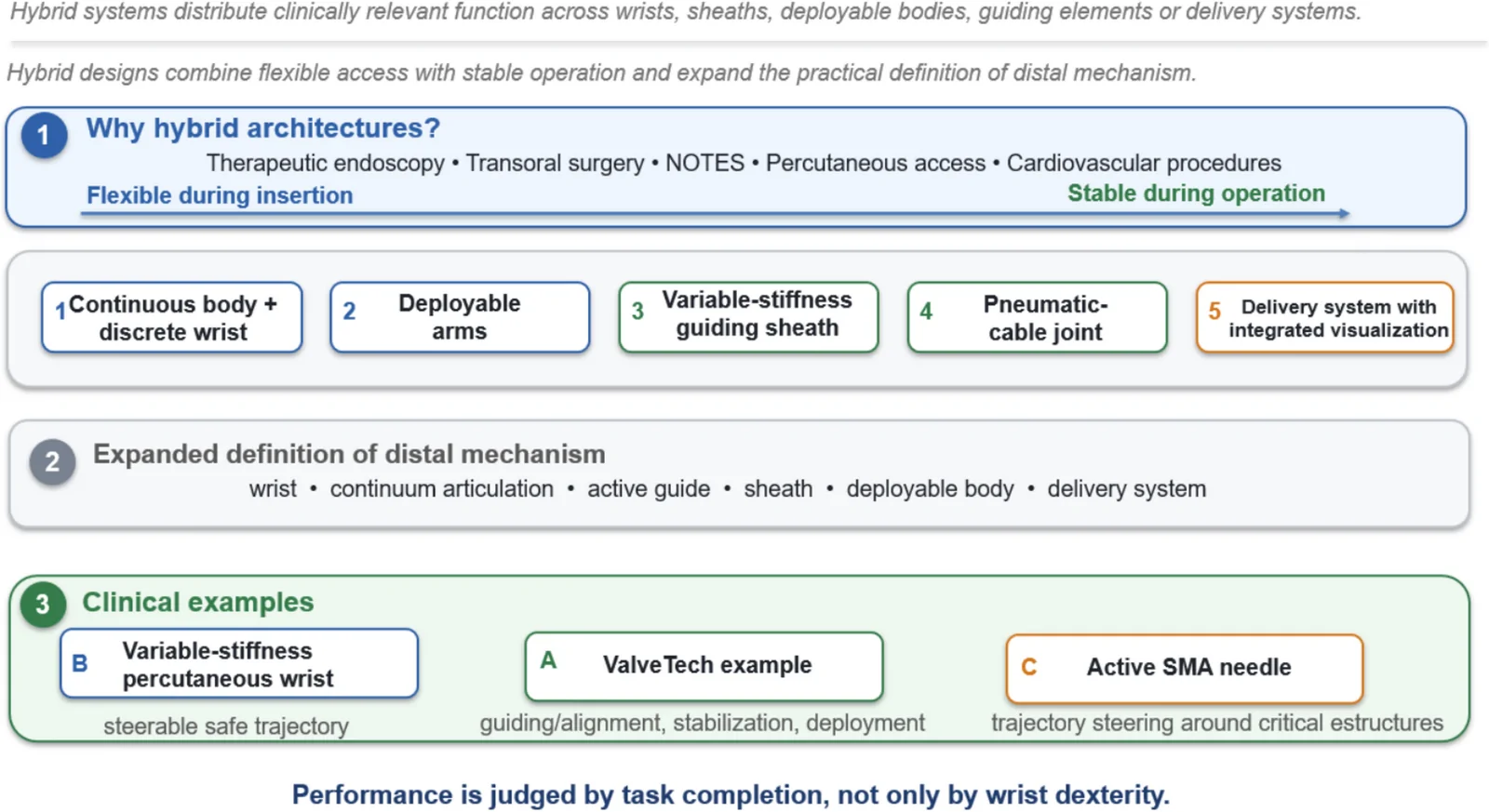
Hybrid distal architectures. Hybrid systems combine access, dexterity, stabilisation, stiffness, sensing or deployment functions

The main risk of hybrid architectures is complexity. Additional mechanisms may improve access, stiffness or dexterity, while increasing diameter, reducing usable lumen, complicating sterilisation, introducing failure modes or making validation more demanding [3, 9, 13, 25, 28, 38]. They should therefore be assessed by whether added complexity produces measurable procedural benefit. Figure 5 illustrates representative hybrid architectures.

### Magnetic actuation as a cross-cutting distal design route

Magnetic actuation requires separate discussion because it changes the relationship between the distal mechanism and the proximal actuation path. Unlike tendon-, cable-, pneumatic- or shaft-driven systems, magnetic approaches can transmit force or torque without a continuous mechanical linkage through the entire access route. This may reduce internal transmission elements and support miniaturisation or steering through complex anatomy, but performance remains coupled to field strength, field gradient, magnet volume, anatomical friction, shape sensing, imaging compatibility, workspace and fail-safe behaviour [36, 37, 77].

Magnetic platforms should be interpreted together with their field-generation infrastructure. Permanent-magnet systems can provide strong fields without continuous electrical power and may be manipulated manually, robotically or through multi-magnet arrangements. Their limitations include source volume, mechanical motion around the patient, distance-dependent gradients, coupled force and torque effects, and the need to avoid unsafe attraction or collision. Electromagnetic coil systems allow faster field modulation, polarity reversal and programmable combinations of uniform fields and gradients, but increase complexity, heating, cooling, power and workspace constraints. MRI-based or hybrid magnetic-navigation approaches may integrate actuation with imaging or localisation, but remain limited by scanner geometry, cost, compatibility and coordination between imaging and actuation. Thus, magnetic actuation should be assessed not only by distal device geometry, but also by force output, moment control, workspace, perception, safety and workflow.

For surgical interpretation, magnetic distal systems should not be treated as a homogeneous family. Three situations are particularly relevant. Magnetically steered continuum catheters or continuum-like mechanisms are mainly evaluated by navigation, shape control, workspace and stability under anatomical constraints [36, 37, 60–64]. Magnetic end effectors or forceps are interpreted through local grasping, orientation, force delivery and tissue interaction [77]. Untethered millimetre- or micro-scale magnetic robots operate at a different structural scale and require separate assessment of localisation, retrieval or biodegradation, imaging visibility, propulsion under flow, small-scale force generation and regulatory translation [58–60, 71, 72]. Magnetic actuation is therefore not a single mechanism family, but a remote actuation strategy whose clinical meaning depends on field source, distal magnetic distribution, structural scale, anatomy and validation context.

Recent literature clarifies these distinctions. Permanent-magnet manipulation of untethered capsule devices has demonstrated multi-DoF control concepts relevant to capsule endoscopy, while magnetic capsule endoscopy also requires localisation, pose estimation and safe interaction control [57, 58, 67]. Teleoperated magnetic endoscopy illustrates workflow implications of robotic magnetic navigation, including field generation by permanent magnets or electromagnetic coils and remote operator control [56]. At catheter and vascular scale, magnetic continuum robots and magnetic soft robots link structural configuration, magnetic distribution, workspace, field generation, anatomical path following and control [60–64]. A multifunctional magnetic catheter robot with triaxial force sensing further shows how distal magnets, sensing and functional instruments can be integrated for palpation, bronchial phantom evaluation and simulated gastrointestinal biopsy tasks [62].

At smaller scales, microrobotics and magnetic soft materials broaden the discussion toward untethered devices, targeted delivery and biohybrid actuation [59, 60, 71, 72]. These works are relevant to remote miniaturisation and non-contact driving, but they should not be used to imply direct equivalence with tethered surgical instruments. In this review, they are cited to clarify scale-dependent opportunities and translation barriers, not to expand the taxonomy beyond distal mechanisms for robotic surgical instruments.

## Miniaturisation as surgical functionality

Distal miniaturisation is not simply a reduction in outer diameter. Cross-sectional space must be distributed among mechanical structure, joints or continuum backbone, transmission elements, working channel, optics, irrigation, insulation, sensing and variable-stiffness components [3, 5, 7–9, 13, 14, 25, 28, 78–81]. Reducing external size may not improve usefulness if it compromises usable lumen, distal force, stability, sensing, vision or task-specific tools [3, 5, 7, 8, 13, 15, 49, 82–88].

Miniaturisation is therefore functional rather than purely dimensional. Devices with similar OD may offer different surgical value depending on whether internal volume supports a working channel, optical pathway, irrigation, force sensing, stiffness-control elements or transmission components [3, 5, 7–9, 13, 25]. Conversely, devices with different dimensions may both be functionally miniaturised if each preserves the capabilities required by its intended procedure.

This distinction is evident across clinical scenarios. In gastrointestinal endoscopy and ESD, miniaturisation must preserve exposure, traction, distal orientation and working-channel compatibility [7, 10, 11, 87]. In transoral surgery and neuroendoscopy, priorities may shift to triangulation, narrow access, protection of critical structures and small-force interaction [8, 13, 77]. In percutaneous or cardiovascular procedures, miniaturisation may be constrained by guidewire compatibility, delivery, deployment or stabilisation [5, 9]. Magnetic catheter robots and robotic endoscopic platforms further show that clinical usefulness depends on how actuation, sensing, lumen and workflow are preserved within the available envelope, not on OD alone [62, 66].

**Fig. 6:**
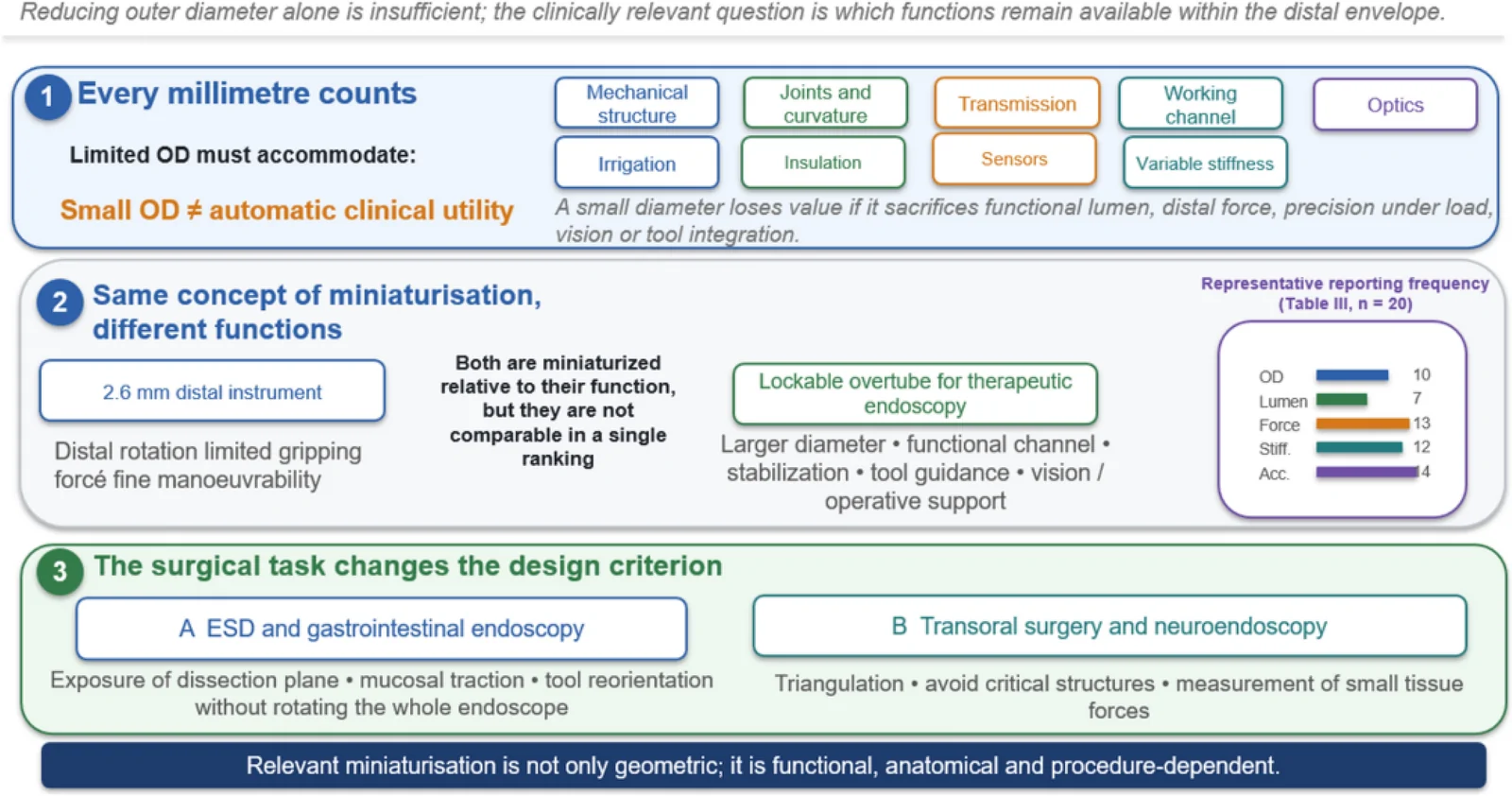
Functional miniaturisation. Reduced OD is meaningful only when lumen, force, stiffness, sensing and task capability are preserved

The relevant question is not simply how small the device is, but which surgical functions remain available within that size. OD should therefore be interpreted together with usable lumen, transmission principle, distal force or load capacity, stiffness, sensing and validation context [3, 5, 7–9, 13, 25, 28]. Figure 6 illustrates miniaturisation as surgical function.

### Recurring trade-off patterns in miniaturisation

Table 3 supports comparison across heterogeneous distal architectures without creating a universal performance ranking. The selected cases are organised by surgical scenario, architecture, structural variables, reported metrics and validation context [3, 7, 9, 13, 28]. OD denotes maximum reported outer diameter. Usable lumen refers to an internal passage for vision, irrigation, instrumentation, cabling or delivery when explicitly described. Functional channel is broader and refers to an available operative channel. When physical lumen and functional channel were not clearly distinguishable, the variable was recorded as NR rather than inferred. DoF denotes degrees of freedom.

**Table 3:** Representative distal mechanisms and procedure-specific variables used for comparative interpretation

Ref.	Representative case and surgical scenario	Distal architecture and transmission	Reported structural variables	Reported functional metrics	Validation context
[3]	Distal hybrid pneumatic/cable-driven continuum joint for flexible gastrointestinal endoscopy	Hybrid continuum joint; cable-driven 2-DoF bending combined with pneumatically regulated variable stiffness	Outer diameter 18 mm; length 110 mm; 2 DoF; nitinol backbone OD 0.8 mm; tendon holes 0.7 mm; tendon diameter 0.5 mm; camera channel 3.8 mm; two instrument channels 3.2 mm; water/air channel 2 mm	Loading capacity 1.50 N in flexible state and 4.80 N in rigid state; rigid-state loading capacity 3.2-fold higher than flexible state; distal deflection 11.87 mm under 5 N payload; deflection under 1 kg distal weight reduced from 41.14 mm at 500 kPa to 35.02 mm at 800 kPa	Characterisation tests, colonoscopy phantom validation and ex vivo organ-pulling experiment
[5]	Wristed percutaneous robot with variable stiffness for pericardiocentesis	Wristed percutaneous mechanism; SMA-spring-based variable-stiffness wrist with cable-driven bending	Outer diameter 4 mm; lumen compatible with 0.5 mm tubing and approximately 200 µm guidewire; flexible wrist length 6 mm; SMA spring backbone; 18-gauge needle; 12-Fr dilator-scale device	Designed to bend the distal tip away from the heart; target curvature >150 m^−1^ to allow 90 degrees bending within less than 10 mm bending length; variable stiffness achieved through SMA temperature change; surface temperature reported below tissue-necrosis threshold	Kinematic and stiffness model verification; gelatin phantom insertion under ultrasound guidance
[36]	Concentric-tube magnetic continuum robot for endovascular intervention	Magnetically actuated continuum/catheter architecture; external magnetic actuation combined with catheter-scale distal steering	Catheter-scale architecture and stiffness-related configuration reported; specific outer diameter, lumen and channel dimensions NR in the consulted extraction	Magnetic steering and stiffness-related behaviour reported; quantitative force, torque, workspace or sensing values NR in the consulted extraction	Bench/prototype validation for potential endovascular intervention
[13]	Cable-driven variable-stiffness manipulator with teeth-engagement structure for transoral surgery	Hybrid cable-driven flexible manipulator with mechanical teeth-engagement variable-stiffness joints	Outer diameter 18 mm; total length 270 mm; bendable segment 110 mm; 8 variable-stiffness joints; CMOS camera channel 2 mm; water/gas channel 1.8 mm; two instrument channels 4.2 mm, compatible with 3.8 mm forceps/electric knife	Stiffness variation ratio up to 84.07-fold; transition time 2.28 s; ex vivo tissue manipulation showed >85% reduction in distal-end positional deviation when stiffness was increased	Bending and stiffness tests, laryngeal phantom experiments and ex vivo tissue experiments
[24]	Soft inflatable cable-driven parallel robot with variable-stiffness end-effector for interventional endoscopy	Hybrid soft parallel mechanism; inflatable structure combined with cable-driven actuation and variable-stiffness end-effector	Structural configuration, actuation principle and end-effector design reported; specific OD, lumen/channel dimensions and DoF NR in the consulted extraction	Motion range, stiffness modulation and load-capacity-related behaviour reported; quantitative values NR in the consulted extraction	Prototype and task-oriented validation for advanced interventional endoscopy
[14]	Miniature spring-based continuum manipulator for minimally invasive surgery	Continuous spring-based cable-driven manipulator with offset neutral bending plane and large central channel	Outer diameter ≤ 3 mm; ID/OD ratio approximately 50%; large central channel; low-cost structure reported as <10 USD per piece	Bending-shape error <0.5 mm; designed for use as activating sleeve or customised controllable instrument; supports instrument delivery through the central channel	Kinematic and statics model validation, peg-transfer teleoperation and porcine tissue laser-ablation experiments
[25]	Robotic guiding sheath with variable stiffness based on conductive graphene and thermoplastic polymer	Hybrid guiding sheath; thermally activated variable-stiffness structure	Sheath geometry and stiffness-control material reported; specific OD, lumen/channel dimensions and DoF NR in the consulted extraction	Stiffness change, response behaviour and guiding performance reported; quantitative values NR in the consulted extraction	Bench/prototype validation
[26]	Phase-change-material-based variable-stiffness sheath for flexible upper gastrointestinal endoscopic robots	Hybrid variable-stiffness sheath; phase-change material inspired by multi-layer wave-spring structure	Sheath structure, material composition and integration constraints reported; specific OD, lumen/channel dimensions and DoF NR in the consulted extraction	Stiffness modulation, thermal response and endoscopic-stability-related behaviour reported; quantitative values NR in the consulted extraction	Bench/prototype validation
[15]	Asymmetric rolling-contact joint for enhanced payload capabilities	Discrete articulated mechanism; asymmetric rolling-contact joint architecture	Joint geometry and distal mechanism configuration reported; specific OD, usable lumen and channel dimensions NR in the consulted extraction	Payload, local orientation, stiffness or load-bearing performance reported; quantitative values NR in the consulted extraction	Bench/prototype and task-oriented validation
[16]	Double-joint catheter robot with wire-reinforced spring backbones	Continuous catheter-like mechanism; wire-reinforced spring backbone with double-joint configuration	Catheter architecture, spring backbone and double-joint arrangement reported; specific OD, lumen/channel dimensions and DoF NR in the consulted extraction	Bending behaviour, analytical model performance, positioning and stiffness/load-related response reported; quantitative values NR in the consulted extraction	Bench/prototype validation
[31]	Miniaturised tendon-driven articulated surgical drill for minimally invasive spine fusion	Discrete articulated distal drill; tendon-driven articulated joints with inner universal-joint torque transmission	Outer diameter 6.4 mm; 2-DoF articulated drilling wrist; joint bend ±45 degrees; inner lumen 2.5 mm; drill-bit collet lumen 2.4 mm	Estimated drilling force approximately 3.6 N and torque approximately 8.1 mN.m; FEA showed dumbbell links support >15 N axial force and universal joint supports >16 mN.m torque transmission	Mechanical performance tests and dVRK-compatible steerable drilling test for minimally invasive spine-fusion scenario
[77]	Magnetically actuated dexterous forceps instruments for neuroendoscopy	Magnetic distal end-effector; permanent-magnet forceps with flexible joints and external magnetic manipulation	Compatible with 3.2 mm neuroendoscopic working channel; previous prototype width 4 mm; Designs A and B provide 3 DoF; Design C provides 2 DoF; magnetic manipulation system opening 190 × 190 mm; usable workspace 60 × 60 × 60 mm	Blocking-force rates of 0.309, 0.880 and 0.351 N/T for the three designs; model prediction error within 14.9%; gripper-closure model mean percent error 11.2%; mean model error 13.1% across grasping behaviours	Bench magnetic-characterisation tests and qualitative pineal-region tumour-resection task in silicone brain phantom
[38]	Deployable arm for natural orifice transluminal endoscopic surgery	Hybrid deployable distal architecture; deployable arm for triangulation and operative separation	Deployable-arm geometry and access constraints reported; specific OD, lumen/channel dimensions and DoF NR in the consulted extraction	Tool separation, operative triangulation and effective angle reported; quantitative values NR in the consulted extraction	Task-oriented validation for NOTES-related use
[7]	Miniature 5-DoF modularised flexible instrument with distal rotation for dual-armed upper gastrointestinal endoscopic robots	Discrete/hybrid miniature flexible instrument; modular 5-DoF architecture with distal rotation	Instrument diameter 2.6 mm; 5 DoF; distal rotation capability reported; lumen/channel constraints reported according to source	Positioning performance, distal loading stiffness, clamping force and ex vivo ESD-related task performance reported; specific extracted numerical values NR in the consulted extraction	Ex vivo/task-oriented validation for upper gastrointestinal endoscopic surgery
[33]	Dynamic hysteresis compensation for tendon-sheath mechanisms in flexible surgical robots	Transmission-focused flexible surgical robot; tendon-sheath actuation without distal perception	Tendon-sheath route and actuation configuration reported; distal geometry, OD and lumen NR in the consulted extraction	Hysteresis compensation, tracking error and distal motion estimation under transmission losses reported; quantitative values NR in the consulted extraction	Bench/control validation
[46]	Force sensing with 1-mm fibre Bragg gratings for flexible endoscopic surgical robots	Distal sensing module; fibre Bragg grating force sensor for tendon-sheath and tendon-driven flexible surgical robots	1 mm FBG bonded to a 3 mm nitinol tube; distal force sensing for tendon-sheath mechanisms; temperature-compensated configuration	Measurement error 0.178 N; sensitivity 34.14 pm/N; tension/compression relationship in tendon-sheath mechanism experimentally verified, with RMSE 0.618 N proximally and 0.332 N distally in the verification setup	Sensor calibration, hysteresis and temperature-compensation tests; demonstration in TSM-driven grasper and tendon-driven continuum robot
[28]	Shape-locking mechanism using double pawl-ratchet system for endoscopic surgery	Hybrid shape-locking overtube; mechanical double pawl-ratchet locking strategy	Outer diameter 19.8 mm; designed for passage through 20 mm natural orifice; total length 112.1 mm; 12 continuum joints; bending capacity ±90 degrees per section	Stiffness approximately 0.88 N/mm; pawl springback coefficient 0.67 N/mm; springback force 2.01 N at 3 mm displacement; locking power requirement 8.93 W; required actuation torque 0.76 Nm and 0.51 Nm	Workspace simulation, locking mechanism test and stiffness tests under vertical loading
[9]	ValveTech robotic approach for minimally invasive aortic valve replacement	Extended distal delivery architecture; flexible manipulator, introducer, valve cartridge, endoscopic vision and stabilisation	Delivery-system geometry, introducer and valve-cartridge integration reported; specific OD, lumen/channel dimensions and DoF NR in the consulted extraction	Valve alignment, stabilisation, deployment capability and surgeon-assessed task performance reported; quantitative values NR in the consulted extraction	Anatomical simulator evaluation with cardiac surgeons
[47]	TCN-based distal force feedback strategy for vascular interventional surgery robot	Sensing/control strategy for vascular interventional robot; data-driven distal force feedback	Catheter/interventional configuration reported; distal sensor geometry, OD and lumen NR in the consulted extraction	Distal force estimation, prediction accuracy and feedback performance reported; quantitative values NR in the consulted extraction	Bench/control validation for vascular intervention
[73]	OpenRST open platform for customisable 3D-printed cable-driven robotic surgical tools	Cable-driven robotic surgical tool platform; customisable 3D-printed distal tools	Tool geometry, cable-driven architecture and customisable structural configuration reported; specific OD, lumen/channel dimensions and DoF NR in the consulted extraction	Motion performance, tool customisation, repeatability or task-level feasibility reported; quantitative values NR in the consulted extraction	Prototype/platform validation

FBG = fibre Bragg grating; GI = gastrointestinal; ID = inner diameter; MISF = minimally invasive spine fusion; SMA = shape memory alloy; TCN = temporal convolutional network; TSM = tendon-sheath mechanism

Values are reported only when explicitly available and verifiable. NR indicates information not reported, unclear or not comparable under a common definition. The table is semi-quantitative, not a standardised benchmark

The 20 representative cases in Table 3 show recurring reporting gaps. Maximum OD or device diameter was reported in 10 of 20 cases (50.0%). Useful lumen or a functional channel was reported in 7 of 20 cases (35.0%). Force-related information, including distal load, payload, force sensing, force prediction or load-related stability, was identified in 13 of 20 cases (65.0%). Stiffness-related information, including structural stability or variable-stiffness behaviour, was reported, modelled or used as the main technical focus in 12 of 20 cases (60.0%). Accuracy-related information, including positioning, tracking, alignment, control or sensing error, was reported in 14 of 20 cases (70.0%).

Workspace was not pooled quantitatively because its definition differed across articulated wrists, continuum segments, catheters and deployable platforms. Validation was also heterogeneous: eight cases were limited to bench, prototype, sensor or control tests; two involved simulated tasks, user studies or surgical training; two used phantom validation without tissue; six involved ex vivo or task-oriented tissue validation; one was cadaveric; and one used an anatomical simulator with surgeons. These categories are descriptive and should not be interpreted as a maturity score.

These percentages apply only to the representative cases in Table 3 and should not be extrapolated to the full search. Variables were counted only when explicitly reported as numerical values or clear functional descriptions. Useful lumen and functional channel were not inferred from device type; force, stiffness, accuracy and workspace were counted only when directly reported, modelled or experimentally evaluated. Unverified values were kept as NR.

To improve evidence granularity, Table 3 distinguishes structural variables, functional metrics and validation context. Reported values such as OD, DoF, sensing-element size, force- or load-related information, stiffness-related behaviour and accuracy-related metrics were retained when available and verifiable. When variables were not reported or could not be clearly distinguished, NR was used to avoid unsupported inference.

## Transmission, sensing and distal reliability

The clinical value of a distal mechanism depends on what reaches the tissue as motion, force, orientation and contact information [46, 47, 73]. A distal architecture may appear capable in isolation, but its usefulness depends on whether intended motion and forces are transmitted through curved or constrained access, and whether tissue interaction is sensed or estimated with enough reliability for the task.

Tendon- and cable-driven instruments allow motors and other bulky components to remain outside the distal end, favouring miniaturisation and reducing the size of the terminal mechanism [18, 73, 81]. However, remote actuation also introduces transmission-related limitations. Pre-tension, cable routing, elasticity, friction, lost motion, backlash, hysteresis and external loading can alter the distal response and reduce motion accuracy, force transmission and tissue-interaction reliability [18, 33, 34, 74, 75]. The transmission pathway should therefore be considered part of the distal design problem, not merely a proximal actuation detail.

Tendon-sheath mechanisms are especially relevant in flexible endoscopy because they transmit motion and force along curved anatomical paths while keeping actuators away from the instrument tip [33, 39–46]. Their main limitation is that sheath curvature and route changes generate friction, tendon elongation, hysteresis, motion loss and force attenuation [33, 34, 39–44]. These effects make distal displacement and force difficult to estimate when the instrument is curved, retroflexed or loaded by tissue interaction [45, 46, 48]. In practical terms, they determine whether a forceps can grasp, retract, expose, cut or stabilise tissue reliably.

Distal sensing addresses the complementary need to make tissue interaction observable. Fibre-optic sensors, triaxial force sensors, miniature six-axis sensors and estimation-based approaches have been proposed to measure or infer distal forces in flexible and miniaturised surgical instruments [46–49]. This is clinically relevant because small unobserved forces may affect tissue safety, dissection quality, grasping reliability or deployment accuracy in confined anatomy.

Recent work on perception and surgical data science reinforces this interpretation. Vision-based endoluminal navigation addresses localisation in narrow, deformable and visually ambiguous luminal anatomy [55], while surgical data science frames perception, context recognition and translation as part of the broader computer-assisted surgery ecosystem [54]. These contributions do not replace distal mechanism design; they show that distal performance becomes clinically interpretable only when mechanical architecture, sensing, perception and workflow are considered together.

Transmission losses may be reduced or compensated through mechanical design, calibration, analytical modelling, anticipatory correction, distal or proximal sensing, indirect estimation or data-driven methods [33, 34, 39, 47, 75]. Model-based approaches are interpretable but may lose accuracy when curvature, routing or load conditions change. Data-driven methods can improve prediction under trained conditions, but their reliability depends on whether the training data cover the anatomical configurations, loads and procedural tasks expected in use [47, 75].

**Fig. 7:**
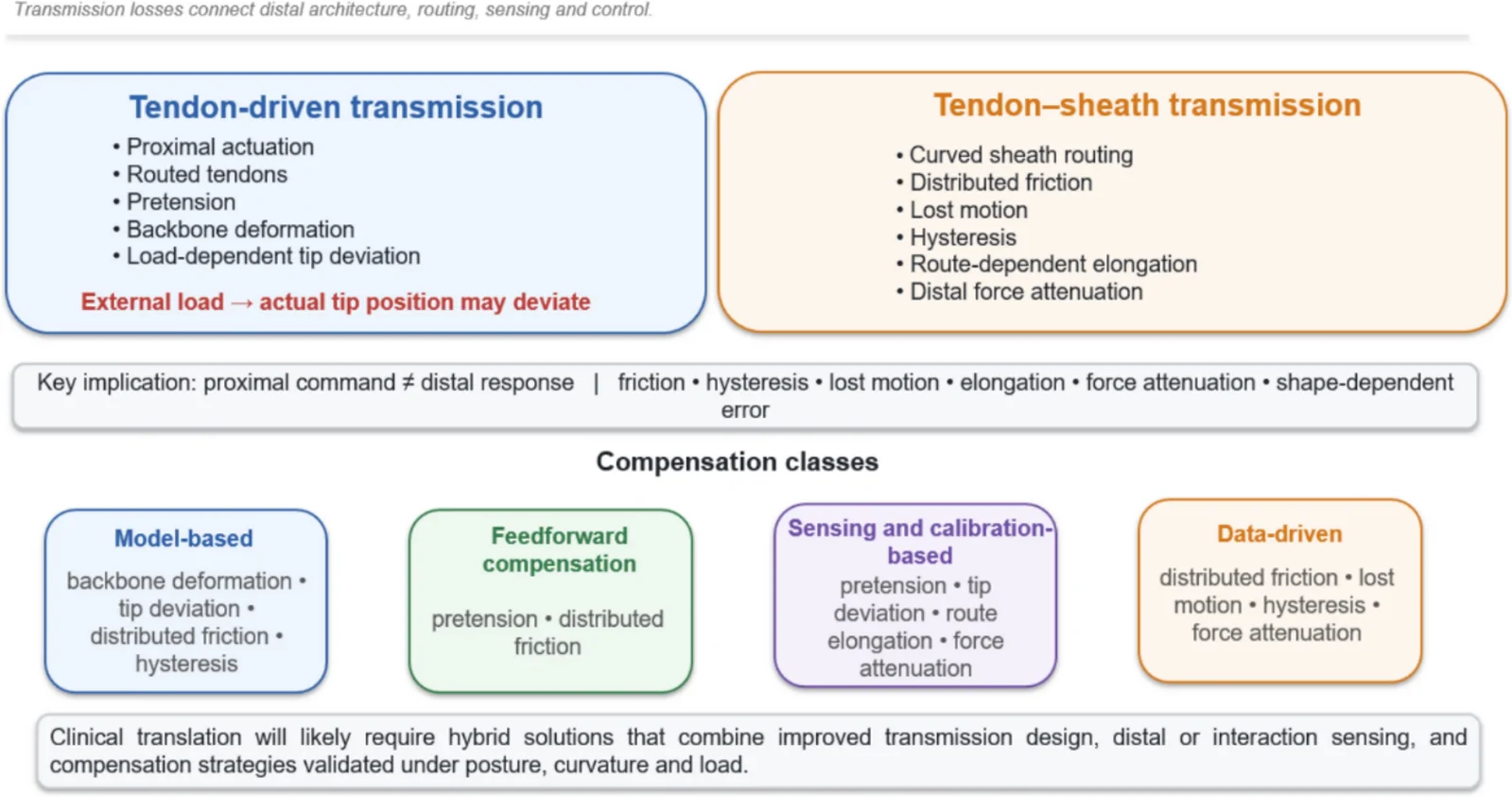
Tendon and tendon-sheath transmission. Friction, hysteresis, lost motion and force attenuation link architecture, sensing and compensation

A clinically robust solution will probably require co-design of architecture, transmission, sensing and control. The mechanism should minimise avoidable losses; sensing should describe actual tissue interaction; and compensation should be validated under representative posture, curvature and loading. Reporting only unloaded accuracy or bench calibration is insufficient when the instrument is intended to grasp, retract, cut, deploy or stabilise tissue in confined anatomy. Figure 7 illustrates these relationships.

## Variable stiffness and shape locking in surgical tasks

Variable stiffness addresses a recurring contradiction in distal surgical instruments: the device should remain compliant during insertion and navigation, but become stable during manipulation, exposure, delivery, dissection or needle guidance [3, 5, 13, 18, 28]. In this review, variable stiffness refers to mechanisms that intentionally change or lock compliance to mediate between access and stable intervention, rather than mechanisms whose stiffness changes only as a passive side effect.

The reviewed literature includes mechanical antagonism, shape-memory-alloy structures, phase-change or thermoplastic materials, fibre, layer, sheet or granular jamming, mechanical shape locking, variable-diameter or variable-stiffness guide sheaths, and hybrid pneumatic-cable continuum joints [3, 5, 13, 18, 25, 26, 28, 29]. These strategies differ not only in stiffness change, but also in integration volume, response time, locking reliability, actuation requirements, sterilisation burden, failure modes and compatibility with the intended workflow [13, 18, 25, 26, 28, 29].

For this reason, variable stiffness should not be evaluated only by maximum stiffness gain. The clinically relevant question is whether the added mechanism improves the intended procedure without unacceptable penalties. Phase-change or thermoplastic sheaths, for example, require attention to thermal response, heating or cooling, latency, safety and endoscopic integration [13, 25, 26]. Jamming-based mechanisms may increase support, but add requirements for sealing, vacuum or pneumatic supply, material arrangement and robustness [18, 29]. Mechanical locking systems must demonstrate predictable engagement and release, load resistance, tolerance to friction or wear, and compatibility with curved anatomical access [28].

The value of stiffness is therefore procedure specific. In transoral surgery, variable stiffness can support atraumatic entry followed by stable tissue exposure or retraction [13]. In pericardiocentesis, insertion stiffness must be combined with distal flexion to redirect the needle away from vulnerable cardiac structures [5]. In upper gastrointestinal endoscopy, variable-stiffness sheaths or hybrid pneumatic-cable joints may stabilise instruments, support tissue manipulation and improve force transmission during therapeutic tasks [3, 15, 25, 26]. In each case, stiffness has value only when interpreted in relation to access route, anatomical risk, dominant task and tissue interaction.

Shape locking is related but distinct. Whereas variable stiffness often implies reversible compliance modulation, shape locking aims to preserve a selected configuration once the instrument has reached or approached the operative site [28]. This can help maintain exposure, alignment or tool stability, but requires reliable engagement, safe release, resistance to expected loads and compatibility with lumen, cleaning, sterilisation and instrument exchange [28].

Thus, variable stiffness and shape locking mediate between flexible access and stable intervention. Their value should be assessed through task-specific metrics such as insertion compatibility, distal stability, load capacity, force transmission, exposure, alignment, response time, reliability, sterilisation and workflow. A high stiffness ratio is meaningful only if it improves the procedure without unacceptable penalties in diameter, safety or usability.

## Procedure-specific performance requirements

Distal mechanisms can be interpreted meaningfully only when the surgical scenario is specified. The same feature may have different value depending on access route, anatomy, dominant task and acceptable tissue interaction. A mechanism suitable for endoluminal navigation may not provide enough stability for suturing; a stiff wrist may aid needle guidance but be unsuitable for atraumatic flexible access. Comparison should therefore be scenario-specific.

In flexible gastrointestinal endoscopy and ESD, distal performance is linked to dissection-plane exposure, mucosal traction, tool orientation and manipulation without rotating the entire endoscope [7, 10, 11, 87]. In transoral surgery, the dominant requirements are narrow natural-orifice access, triangulation, collision avoidance, tissue exposure and stable manipulation [1, 6, 13]. In NOTES and therapeutic endoluminal surgery, flexible entry must be combined with distal deployment, operative triangulation and maintenance of an effective working angle [38].

Percutaneous and needle-guidance procedures impose different constraints: the access path is narrow and direct, but risk is concentrated around the needle or distal tool. Relevant variables include tip control, steering accuracy, insertion stiffness, distal flexion and anatomical or phantom validation [5]. In vascular and endovascular intervention, catheter-scale dimensions, curved anatomy, guidewire compatibility and atraumatic navigation make distal force estimation, route-dependent transmission and contact-force control especially important [36, 37, 47, 89–92]. In neuroendoscopy and small-cavity surgery, precise small-force interaction, visibility and compatibility with delicate anatomy may matter more than large workspace or high payload [8, 77].

These examples show that no single metric can serve as a universal benchmark. OD, DoF, workspace, stiffness, distal force, sensing accuracy and validation level must be interpreted in relation to the surgical task. Table 4 summarises surgically interpretable requirements and comparison criteria.

**Table 4:** Surgically interpretable requirements and comparison criteria for distal mechanisms in robotic surgery

Scenario	Dominant clinical function	Relevant metrics	Interpretive caution
Flexible gastrointestinal endoscopy and ESD	Exposure of the dissection plane, mucosal traction, tissue manipulation and distal tool orientation	OD, usable lumen or working channel, distal rotation, grasping force, traction capability, positioning accuracy, distal stability and ex vivo task performance	A smaller OD is not sufficient if traction, exposure, stability or working-channel compatibility are compromised. Metrics should be interpreted in relation to the endoscopic task rather than instrument size alone
Transoral robotic surgery	Access through a constrained natural orifice, triangulation, collision avoidance, tissue exposure and stable manipulation	Distal dexterity, triangulation, instrument separation, collision avoidance, variable stiffness, load capacity, workspace within the oral/pharyngeal corridor and cadaveric or task-oriented validation	Workspace alone may be misleading if it does not account for insertion route, anatomical constraints, visualisation and instrument-instrument collisions
NOTES and therapeutic endoluminal surgery	Flexible entry, distal deployment, operative triangulation and maintenance of an effective working angle	Deployability, tool separation, effective angle between instruments, stability, payload, working-channel use and phantom/ex vivo task performance	Dexterity should not be interpreted independently of access route, deployment sequence, endoscope compatibility and ability to maintain a stable operative triangle
Percutaneous and needle-guidance procedures	Controlled insertion, distal redirection, trajectory correction and risk reduction near vulnerable structures	Needle or instrument diameter, insertion stiffness, distal flexion, steering accuracy, trajectory control, tip stability and phantom or anatomical-model validation	High stiffness may support insertion but can increase anatomical risk if distal redirection or fail-safe behaviour is not demonstrated
Vascular and endovascular intervention	Atraumatic navigation through curved vascular anatomy, distal steering and force-aware interaction	Catheter-scale diameter, steerability, shape control, force estimation, distal force feedback, route-dependent transmission behaviour and bench/interventional validation	Small forces can be clinically relevant. Force estimation and navigation metrics should be interpreted with respect to vessel-wall safety and route-dependent transmission losses
Neuroendoscopy and small-cavity surgery	Miniaturised access, controlled manipulation and safe interaction in highly confined spaces	Tip size, distal dexterity, small-force sensing, grasping or tool action, positioning accuracy, sensor integration and task-specific validation	Large workspace or high payload may be less relevant than precise small-scale interaction, minimal tissue contact and compatibility with delicate anatomy
Cardiovascular delivery and deployment systems	Guided delivery, distal alignment, stabilisation and controlled deployment of devices	Delivery pathway, alignment accuracy, stabilisation, deployment success, visualisation, surgeon-assessed usability and anatomical-simulator validation	The clinically relevant distal mechanism may include the introducer, cartridge, visualisation and stabilisation system, not only a conventional articulated end effector

## Validation maturity and clinical translation

A recurring limitation in the literature is the gap between mechanical demonstration and clinically interpretable validation. Many studies characterise kinematics, workspace, stiffness, force, hysteresis, locking behaviour, sensing error or tracking accuracy through bench tests, prototype evaluations, sensor tests or control experiments [28, 33, 34, 46–49, 73, 93]. These studies clarify mechanism behaviour, but do not automatically demonstrate surgical usefulness. A distal mechanism must also preserve its intended function under anatomical constraints, tissue interaction, visualisation requirements and workflow limitations.

Other studies move closer to surgical interpretation by using phantoms, ex vivo tissue, anatomical models, cadavers, animals or surgeon evaluations [3, 7, 9, 13, 15, 24, 94]. These contexts provide different evidence: phantoms test access, navigation, alignment and workflow; ex vivo tissue evaluates traction, cutting, grasping or dissection; and cadaveric, animal or surgeon-involved studies add anatomical or procedural realism, although they do not by themselves establish clinical effectiveness.

Recent systems further illustrate this heterogeneity. Magnetic endoscopy and capsule-navigation studies show that remote actuation must be evaluated with localisation, workspace and interaction-safety constraints [56, 57, 67]. Magnetic continuum and catheter-based systems provide phantom, ex vivo, in vivo or task-oriented evidence for vascular navigation, palpation, biopsy support and telemanipulated intervention [62–64, 70]. Vision-based endoluminal navigation contributes dataset-based evidence for localisation in visually ambiguous anatomy, whereas ROSE provides in vitro and ex vivo evidence for colonoscopic locomotion and anchoring [55, 66]. These examples reinforce that validation maturity should be described by task, environment and measured function, not by a single generic hierarchy.

The most informative studies explicitly link distal design with surgical task. Early flexible endoluminal robotic systems, including prototype robotic endoscopes, MASTER and EndoMaster, provided task-level evidence for bimanual distal manipulation in ESD, NOTES and transoral applications [6, 84–86]. Deployable arms in NOTES show that distal separation, triangulation and effective instrument angle are key determinants of procedural capability [38]. The 2.6-mm modular flexible instrument links miniaturisation with five-DoF distal motion, distal rotation, calibrated positioning, loading stiffness, clamping force and ex vivo ESD testing [7]. ValveTech extends this logic to cardiovascular delivery, where performance is assessed through alignment, stabilisation and deployment in an anatomical simulator with cardiac surgeons rather than through kinematic dexterity alone [9].

Validation maturity should therefore not be interpreted as a simple hierarchy in which one experimental stage is always superior. Bench tests quantify mechanism behaviour, but may not reveal access, workflow or tissue-interaction limitations. Phantoms and anatomical models clarify navigation, deployment and user interaction, but may not reproduce tissue properties. Ex vivo tissue can reveal manipulation and cutting behaviour, but may not fully represent in vivo constraints. Cadaveric, animal or early clinical evaluations provide important feasibility evidence, but do not replace mechanism-level characterisation. A mature pathway should accumulate evidence across mechanism performance, task-level function, anatomical feasibility and, eventually, clinical safety and efficacy.

In this review, validation maturity is descriptive rather than a formal risk-of-bias or study-quality score. The classification identifies the type of evidence reported, but it does not assign equal evidentiary weight to all studies. Sample size, number of repetitions, control conditions, loading protocols, measurement methods, statistical analysis, surgeon involvement, tissue realism and task fidelity remain critical for judging whether validation is adequate for a specific surgical claim. Low-maturity concept demonstrations should therefore not be interpreted as equivalent to task-level, tissue-based, cadaveric, animal or surgeon-evaluated studies. Validation adequacy was interpreted according to the match between experimental setting, measured variables, task fidelity, anatomical realism and claim strength.

Clinical translation is not only progression to human trials. Before that stage, a distal mechanism must demonstrate compatibility with workflow, visualisation, instrument exchange, cleaning or sterilisation, fail-safe behaviour and training [9, 28]. A prototype that performs well on the bench may still be difficult to adopt if it requires complex calibration, long set-up, unrealistic routing, fragile components or difficult sterilisation. Translational readiness therefore requires evidence that the mechanism can function within the procedural ecosystem.

Validation should be scenario- and function-specific. ESD mechanisms should be evaluated by tissue exposure, traction, distal orientation, access to the dissection plane and endoscope compatibility. Transoral mechanisms require assessment of oral and pharyngeal access, triangulation, collision avoidance and stable tissue manipulation. Percutaneous or cardiovascular devices should report guidance, deployment, contact forces and risk reduction. Neuroendoscopic instruments require delicate force interaction, visibility and confined-space control. Future studies should report not only what the mechanism can do mechanically, but also where, under what access conditions and for which surgical task.

## Practical recommendations for authors, reviewers and designers

Distal mechanisms are difficult to compare when key information is missing or disconnected from procedural context. Diameter, DoF, stiffness, workspace, force and sensing accuracy become clinically meaningful only when linked to surgical scenario, access route, tissue interaction and validation conditions. Isolated mechanical values should therefore not be treated as universal indicators of usefulness.

Authors should first define the intended surgical function before presenting a mechanism as an improvement. For navigation, relevant variables include access geometry, conformability, shape estimation and tissue contact. For traction or exposure, distal force, stiffness, stability and orientation are central. For cutting, suturing, deployment or needle guidance, authors should report task-specific accuracy, load response, fail-safe behaviour and validation conditions.

A second recommendation is to describe the distal mechanism as an integrated functional unit. Distal architecture, transmission path, usable lumen, sensing capability, stiffness behaviour and validation environment should be reported together, especially in flexible or endoluminal systems where friction, hysteresis, force attenuation and shape uncertainty determine whether the intended distal action reaches tissue. Comparative claims such as “more dexterous,” “more flexible,” “smaller,” “stiffer” or “more stable” should include an operational definition, test condition and procedural interpretation.

Table 5 proposes a minimum reporting core for distal mechanisms in robotic surgical instruments. The aim is not to impose a universal benchmark, but to make comparisons clearer across heterogeneous surgical scenarios.

**Table 5:** Minimum reporting core for distal mechanisms in robotic surgical instruments

Reporting domain	Minimum item to report	Why it matters
Intended surgical use	Surgical scenario, access route and dominant task	Defines the procedure-specific function, such as navigation, exposure, traction, cutting, suturing, deployment or needle guidance
Distal architecture	Discrete, continuous, hybrid or extended architecture	Clarifies how motion, curvature, stability and tissue interaction are generated
External dimensions	Maximum OD or device envelope	Supports assessment of anatomical compatibility and miniaturisation constraints
Usable lumen or functional channel	Working channel, guidewire lumen, optical pathway, irrigation and suction pathway or delivery channel, when applicable	Prevents OD from being interpreted as full clinical capacity
DoF and motion range	DoF, bending range, rotation, articulation or deployment capability	Describes distal motion in relation to the surgical task
Transmission principle	Tendon, cable, tendon-sheath, pneumatic, magnetic, hydraulic, mechanical linkage or hybrid transmission	Indicates expected losses, hysteresis, backlash, force attenuation and control limitations
Distal force or load capacity	Grasping force, payload, traction force, insertion force, distal load or force-estimation range	Indicates whether the mechanism can perform the intended interaction or deployment task
Stiffness definition	Direction, posture, loading condition and test method	Avoids comparing stiffness values measured under incompatible conditions
Sensing capability	Type, location, range and calibration of force, shape, position or contact sensing	Defines whether tissue interaction and distal state can be observed or estimated
Accuracy and repeatability	Positioning, tracking, alignment, repeatability or task-specific error	Links mechanical performance with surgical precision
Workspace or reachability	Workspace definition, anatomical constraint or target reachability	Prevents workspace from being interpreted without access-route constraints
Validation context	Bench, phantom, ex vivo tissue, anatomical model, cadaveric, animal, simulator, surgeon evaluation or clinical use	Indicates how close the evidence is to clinically interpretable performance
Workflow and translation factors	Instrument exchange, visualisation, cleaning, sterilisation, fail-safe release and training requirements	Helps assess integration into real surgical workflow
Missing information	Use NR when information is not reported or not clearly distinguishable	Avoids unsupported inference and improves comparability

The reporting core in Table 5 may also help peer review. Reviewers should not expect every mechanism to outperform previous designs in every metric. Instead, they should assess whether reported variables explain the intended surgical role, allow comparison with relevant alternatives and show appropriate validation maturity. For designers, the reporting core can serve as a checklist to identify the variable most likely to limit surgical performance: access, lumen, force, stiffness, sensing, stability, sterilisation, workflow or validation.

For surgeons and clinical evaluators, the reporting core helps translate engineering metrics into operative relevance. It clarifies whether a distal mechanism supports the intended task, fits the access route, preserves workflow and safety requirements, and has been validated under clinically interpretable conditions. The goal is not to identify one universally superior architecture, but to support scenario-sensitive comparison and translation.

**Fig. 8:**
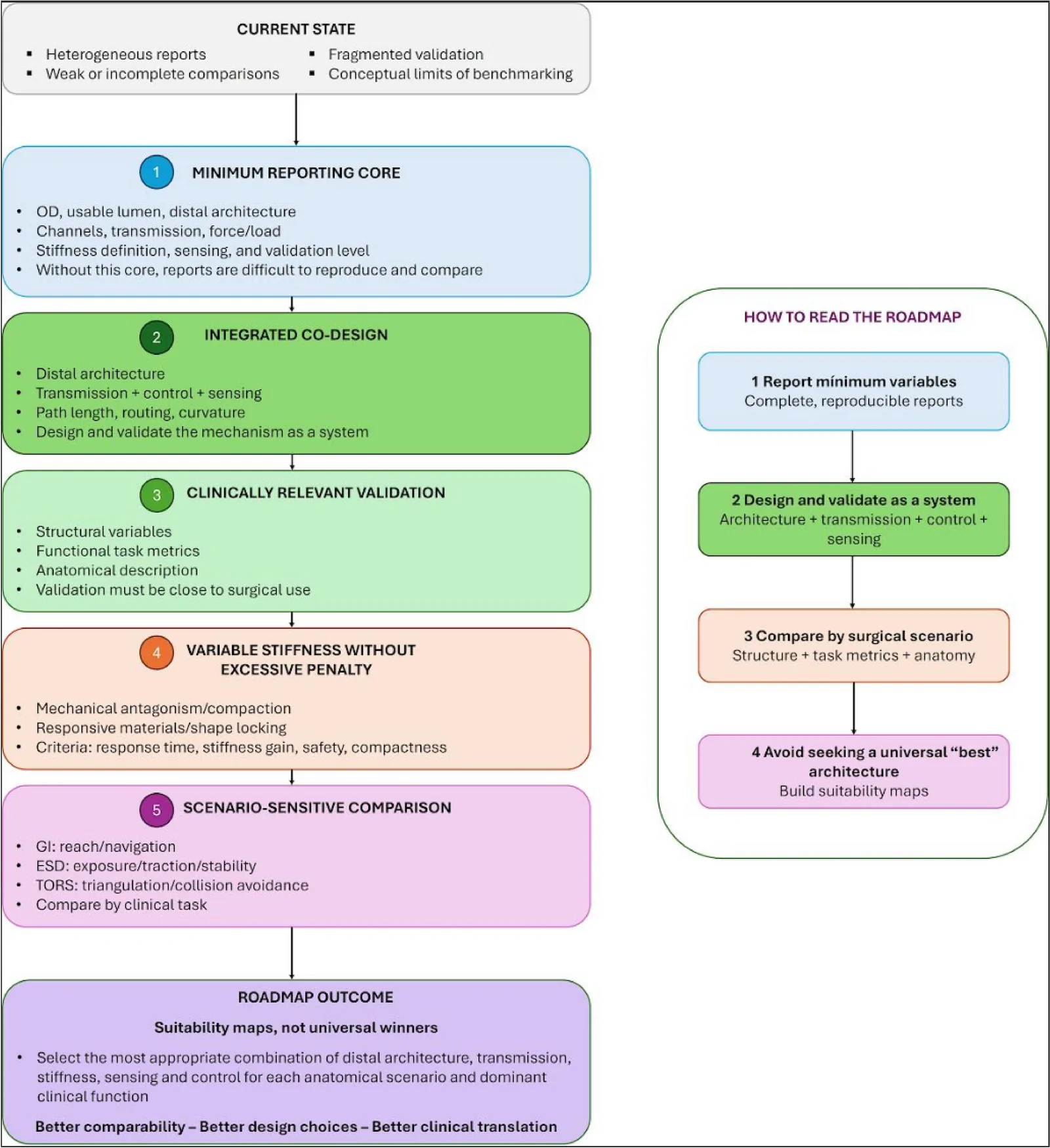
Reporting and validation roadmap. Minimum reporting, integrated co-design and scenario-sensitive validation support interpretable comparison

Figure 8 summarises the proposed roadmap for reporting, validation and scenario-sensitive comparison.

## Limitations of the review

First, this was designed as a structured narrative review rather than a PRISMA-compliant systematic review or meta-analysis. Its purpose was to organise and interpret technical and clinically relevant evidence on distal mechanisms, miniaturisation, transmission, stiffness, sensing and validation, not to provide exhaustive quantitative synthesis of clinical outcomes. Conclusions should therefore be read as design and reporting guidance, not pooled estimates of efficacy, safety or superiority.

Second, the search used IEEE Xplore, PubMed and Web of Science, which are appropriate for a technical-clinical review of robotics, mechatronics, instrumentation, sensors, control and medical robotics. However, Scopus and clinical trial registries were not included. Some clinical reports, case series, anatomical studies, surgical feasibility studies or early translational evidence may therefore have been missed, and the review should not be interpreted as a comprehensive assessment of clinical adoption or therapeutic effectiveness.

Third, the reviewed studies were highly heterogeneous in surgical scenario, access route, architecture, scale, actuation, transmission, sensing, validation environment and metrics. Direct quantitative comparison and pooled analysis were therefore inappropriate. Reported variables were also inconsistent: OD, usable lumen, distal force, stiffness, accuracy, workspace and validation level were not reported uniformly. Unclear or missing information was recorded as NR, avoiding unsupported inference but limiting cross-study comparison.

Because the review was not designed as a formal systematic review, it did not conduct a quantitative risk-of-bias assessment or formal validation-quality score. Validation maturity was interpreted qualitatively, and bench demonstrations, control simulations, ex vivo tests, cadaveric work, animal studies and surgeon-involved anatomical simulation were not treated as equivalent evidence.

Fourth, the selection flow could not be reconstructed as a complete record-level database. Article-level exclusion reasons, full-text retrieval status and duplicate-handling information were not documented for every retrieved record and were therefore not reconstructed retrospectively. This preserves traceability but limits systematic-review reproducibility.

Finally, this review focuses on distal mechanisms and their technical-clinical interpretation. It does not fully assess regulatory pathways, cost-effectiveness, surgeon training, hospital adoption, patient-reported outcomes or long-term performance. It is also not intended as an exhaustive systematic map of all research teams, all medical imaging literature or all micro/nanorobotic drug-delivery systems. Additional literature on magnetic actuation, surgical vision, magnetic continuum robots, microrobotics and continuum-robot modelling was included only when it clarified distal robotic function, surgical interpretability or translational constraints. Future work should include Scopus, clinical trial databases and procedure-specific clinical literature to evaluate safety, usability and translational outcomes.

## Conclusions

Distal mechanisms bring together many central challenges in robotic surgical instruments: anatomical access, local dexterity, tissue interaction, remote transmission, stiffness control, sensing and validation. The reviewed literature does not show that any distal architecture is universally superior. Discrete articulated, continuous, hybrid and extended mechanisms offer different trade-offs among precision, load capacity, conformability, stability, miniaturisation and procedural complexity.

Miniaturisation should be understood as functional rather than purely dimensional. A smaller instrument is useful only if it preserves lumen, distal force, stiffness, sensing, visualisation and task-specific capability. Transmission is equally important: tendon, cable and tendon-sheath systems support remote actuation and miniaturisation, but friction, hysteresis, lost motion, elongation and force attenuation determine whether distal action reaches tissue.

Variable stiffness and shape locking can balance flexible access with stable intervention, but they may add penalties in diameter, response time, thermal or pneumatic requirements, sterilisation, reliability and workflow integration. Stiffness should therefore not be evaluated only by maximum gain, but by how much it contributes to a specific surgical task under realistic access and loading conditions.

Distal mechanisms should be compared through task-dependent interpretation rather than isolated mechanical indicators. OD, DoF, workspace, stiffness, distal force, sensing accuracy and validation level are useful only when connected to anatomical route, tissue interaction, workflow and task requirements. Progress will depend less on one best architecture than on coherent combinations of mechanism, transmission, stiffness, sensing and control for each surgical scenario.

A minimum reporting core is needed for meaningful comparison. Authors should report distal architecture, OD, usable lumen or functional channel, transmission principle, force or load capacity, stiffness definition, sensing capability, accuracy, validation context and workflow constraints. Consistent reporting would help distinguish mechanical feasibility, task-level usefulness and translational readiness.

## Data Availability

Screening data and supplementary tables derived from available inputs are included as supplementary material. No new datasets were generated during the preparation of this review.

## Declarations

### Declaration on the use of artificial intelligence-assisted tools

Artificial intelligence-assisted tools were used to support language editing, structural refinement, consistency checking and preliminary organisation of non-final draft material. These tools were not used as autonomous agents for literature selection, eligibility decisions, data extraction, technical interpretation, reference verification or final scientific judgement. No artificial intelligence tool was considered an author. The authors reviewed, corrected and approved all decisions related to literature selection, technical interpretation, descriptive calculations, table content, figure content, reference verification and final drafting. The authors assume full responsibility for the accuracy, integrity, originality and scientific content of the manuscript.
